# Muscle Tenderness and Psychiatric Comorbidity: A Vicious Cycle in Migraine Chronicization

**DOI:** 10.3389/fneur.2014.00148

**Published:** 2014-08-06

**Authors:** Eugenia Rota, Franco Mongini

**Affiliations:** ^1^Neurology Unit, Guglielmo da Saliceto Hospital, Piacenza, Italy; ^2^Headache-Facial Pain Section, Department of Clinical Pathophysiology, University of Turin, Turin, Italy

**Keywords:** headache, migraine, tension-type headache, muscle tenderness, chronicization

Migraine is a highly prevalent, disabling disorder, and has a remarkable feature, comorbidity with other neurological and psychiatric diseases. A relevant number of general population studies (mainly cross-sectional, but also prospective) and clinical studies have unquestionably demonstrated the association between migraine and psychiatric disorders (axis 1 of the DSM) (1–6), whilst the mechanisms underlying such comorbidity are still to be fully clarified. Most of the evidence supports the association between migraine, above all chronic and with aura, on the one hand, and major depression and anxiety disorder, on the other, although even bipolar disorder and obsessive compulsive disorder have been linked to migraine. Patients with migraine have a two/threefold increased lifetime relative risk of major depression, compared to non-migrainous subjects (1–3).

This comorbidity between affective disorders and migraine seems to result from bidirectional influences, where each disorder increases the risk for first onset of the other. Indeed, subjects suffering from anxiety and a combination of anxiety disorders and major depression are more likely to have migraine (2, 3). Furthermore, in migraine patients, the co-occurrence of personality changes and depression, although does not appear to influence the response to treatment at short term, may affect the headache natural history in the long term, independently from the pain characteristics at the baseline, as demonstrated by a longitudinal study (7).

Such bidirectional relationship between migraine and affective disorders may be underpinned by shared genetic or epigenetic factors able to increase the risk of both conditions (8). In a recent study (9), a new polygenic (genetic risk) score analysis was applied to investigate the mechanisms underlying the genetic overlap of migraine and major depressive disorder. The subgroup of individuals with comorbid major depressive disorder and migraine were genetically most similar to major depressive disorder patients, so that the authors state that: “in at least a subset of migraine patients with major depressive disorder, migraine may be a symptom or consequence of major depressive disorder” (9). Along with shared genetic features, other common pathophysiological factors, such as neuronal excitability and vascular endothelial dysfunction (10, 11), might play a role in the relationship between migraine and psychiatric disorders. They are poorly understood, making it a priority for future research.

Growing evidence suggests that psychiatric comorbidity may be a risk factor for migraine chronicization (6). A shared dysfunction of the serotoninergic neurotransmission, medication overuse, and predisposing personality traits have been hypothesized to underpin this role of affective disorders in promoting the progression from episodic to chronic migraine, although the mechanisms involved are still to be fully clarified (12, 13).

Although often underestimated, increased tenderness at palpation of the pericranial and cervical muscles is common in migraine patients. It is generally agreed that muscle tenderness is frequently associated to tension-type headache, as provided by the Headache Classification Committee of the International Headache Society (IHS) (14), where infrequent, frequent and chronic tension-type headache can be associated with increased pericranial muscle tenderness. Patients with tension-type headache, compared with asymptomatic subjects, showed higher tenderness of the pericranial muscles and EMG activity of the temporal muscle (15, 16). Moreover, muscle tenderness was found to increase significantly with increasing frequency of tension-type headache (15).

While limited and conflicting information is available on muscle tenderness in migraine patients, some data suggest that muscle tenderness could be involved in migraine (17–19). A general neck–shoulder muscle hyperalgesia was detected in both chronic tension-type headache and unilateral migraine patients (20). In a recent population study (21), neck pain prevalence was higher in subjects with primary headache, mainly in those with coexistent migraine and tension-type headache, and myofascial tenderness was significantly increased in individuals with neck pain (21).

Considering the aforementioned relationship between migraine and psychiatric disorders, a study investigated the extent to which, in headache patients, muscle tenderness could relate to such disorders (22). Muscle palpation of pericranial and cervical muscles were carried out in a large sample of patients with episodic migraine, chronic migraine, episodic tension-type headache, chronic tension-type headache, and episodic migraine and tension-type headache together. For each patient a psychological assessment on the axis 1 of the DSM-IV was performed. Anxiety and depression were significantly associated to chronic migraine. Moreover, a positive relationship was found between muscle tenderness and psychiatric disorders in patients with episodic migraine. Indeed, in the group with episodic migraine, muscle tenderness scored consistently higher in patients with anxiety or anxiety and depression as compared to those without such disorders (22).

Other research has demonstrated that, in tension-type headache, continuous nociceptive input from peripheral tender muscles may trigger central sensitization, finally leading to headache chronicization (15, 23, 24).

Taking into account all this evidence together, we hypothesize that in patients with episodic migraine, who also have anxiety or anxiety and depression combined, the increased level of muscle tenderness in the head and in the neck might facilitate the evolution into chronic migraine (25).

Indeed, the higher muscle tenderness in those migraine patients with psychiatric disorders might well be the missing link, at least one of the factors that could lead to a vicious circle in the natural history of migraine and promote its evolution into the chronic form. Hence, from a clinical perspective, it is mandatory to diagnose and appropriately treat comorbid psychiatric diseases in migraineurs, and, similarly, to recognize increased muscle tenderness, as such conditions may negatively affect migraine course and increase the migraine-related disability.

Hopefully, further longitudinal studies will help to clarify the complex interplay between psychiatric comorbidity and muscle tenderness and their role in the natural history of migraine.

## Conflict of Interest Statement

The authors declare that the research was conducted in the absence of any commercial or financial relationships that could be construed as a potential conflict of interest.
